# Supplementary material for: How heat propagates in liquid 3He
Source: Nat Commun. 2024 Feb 27;15:1771. doi: 10.1038/s41467-024-46079-0 (PMC10899593; doi:10.1038/s41467-024-46079-0)
Supplement: Supplementary file 1 — Supplementary Information [file 41467_2024_46079_MOESM1_ESM.pdf]

Supplementary Information for "How heat propagates in liquid ^{3}He"

Supplementary notes

Supplementary Note 1: Departure from Fermi liquid behavior due to spin fluctuations

Several early attempts to explain the non-Fermi liquid behavior observed in ^{3}He invoked spin fluctuations. Since the exchange energy of spins is in the range $\approx$ 1 mK, this approach is not sufficient to explain the non-Fermi liquid behavior seen in a temperature range, which is at least two orders of magnitude larger. Nuclear magnetic relaxation measurements have found that the exchange energy of spins is J=0.002 K when the molar volume is 24.6 cm^3/mol [1]. Specific heat measurements find that J=0.00085 K, when the molar volume is 24.45 cm^3/mol [2]. These numbers are to be compared to the Fermi ($\approx$ 2 K) and Debye energy ($\approx$ 20 K). There is no detectable evidence of a role played by spin fluctuations in heat transport (either as conductors of heat or as scattering centers) when the temperature exceeds 0.1 K.

Supplementary Note 2: Lower bounds on thermal diffusivity and viscosity in classical liquids

In liquid and supercritical states of matter, thermal conductivity and thermal diffusivity are strongly system-dependent and can change by many orders of magnitude depending on temperature. However, there is a universal theoretical lower bound of thermal diffusivity, α_{min}, defined by [3]:

$$\alpha_{min} = \frac{1}{4\pi}\frac{\hbar}{\sqrt{m_e m}} \tag{S1}$$

where m_e is the electron mass and m is the molecule mass.

The lower bound corresponds to the minima seen in the Supplementary Figure 1. The minimum itself is due to the dynamical crossover between the low-temperature liquidlike regime where each particle undergoes both oscillatory and diffusive motion and the high-temperature gaslike regime where the oscillatory component of motion is lost and only the diffusive component is present [3–5]. The value of thermal diffusivity at the minimum can be evaluated by equating either the phonon mean free path in the liquidlike regime or the particle mean free path in the gaslike regime to the interatomic separation. Then, the value at the minimum is related to only two parameters in condensed matter:

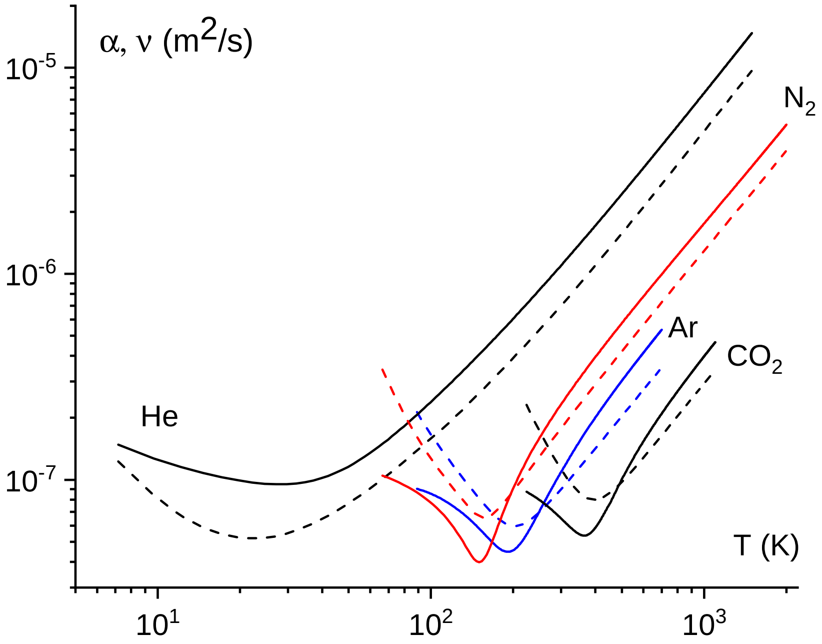

Supplementary Figure 1: Minima of thermal diffusivity and kinematic viscosity in classical liquids. Experimental curves of thermal diffusivity (solid lines) and kinematic viscosity (dashed lines) for ^{4}He (20 MPa), N_2 (10 MPa), Ar (20 MPa), and CO_2 (30 MPa). .

interatomic separation and Debye vibration frequency. Relating this length and energy scale to the fundamental physical constants using Bohr radius and Rydberg energy gives Eq. (S1) [3, 5].

This theoretical minimum in thermal diffusivity, α_{min}, coincides with a minimum in the kinematic viscosity ν_{min} [4]. As seen in Figure 1 of the main text, the experimental data confirms that the two minima are close to each other.

Supplementary Note 3: The dimensionless fermion-fermion collision cross section

The relevance of Planckian bound when the temperature is more than one order of magnitude below the degeneracy temperature of fermionic quasi-particles is driven by the unusually large fermion-fermion scattering rate in ^{3}He [8].

The dimensionless cross section of fermion-fermion scattering, $\zeta = \frac{\hbar E_F}{\tau_\kappa (k_B T)^2}$, in ^{3}He is as large as $\sim$ 35 at zero pressure and shoots up to $\sim$ 60 at the melting pressure. In metals, this cross section is often smaller [9]. When electrons are weakly scattered, ζ seldom exceeds unity. But in strongly correlated metals, it is as large as 10. In $La_{1.67}Sr_{0.33}CuO_4$ cuprate, ζ is almost as large as in ^{3}He [8].

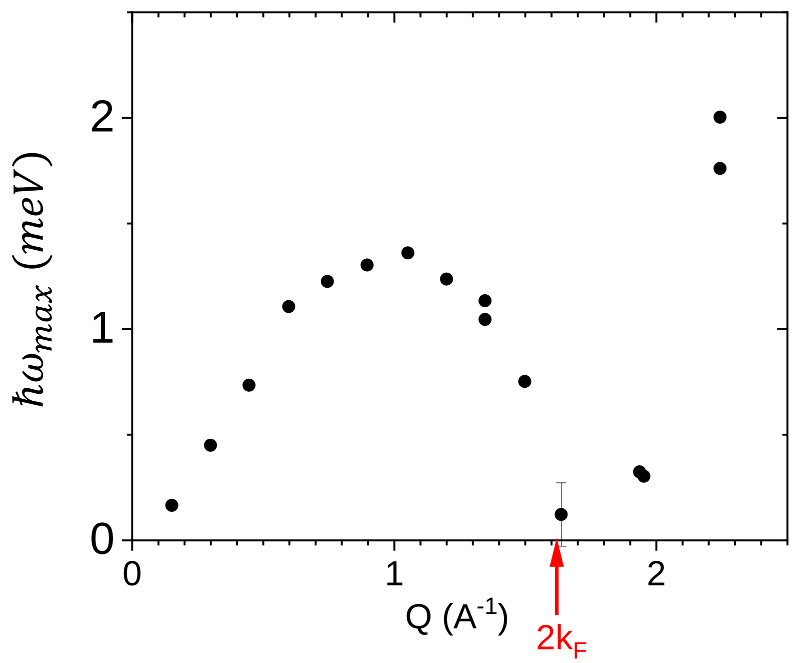

Supplementary Figure 2: Phonon dispersion according to inelastic X-ray scattering experiments. Acoustic dispersion curves for normal liquid ^{3}He at 1.1 K [6, 7]. The red arrow points to $Q = 2k_F$=1.59Å^{-1}.

Supplementary Note 4: Thermal conductivity in the Landauer picture

Landauer pictured conduction as the transmission of a wave attenuated over a distance. When the wave is a ballistic quasi-particle, the attenuation distance (or the mean-free-path) is set by the finite size. One virtue of this approach is the transparency of the presence of fundamental constants and context-dependent length scales in setting the expected magnitude and temperature dependence of a transport coefficient.

The Landauer formalism leads to the following expression for the electrical conductivity of a two-dimensional metal whose Fermi surface is a circle of radius k_F and its mean-free-path ℓ:

$$\sigma^{2D} = \frac{e^2}{h} k_F \ell \tag{S2}$$

This is because the number of conducting mode is $2 \times 1/2 \times \frac{2\pi}{\lambda_F} = k_F$ (2 for spin degeneracy, $\frac{1}{2}$ for averaging a vector in two dimensions, and 2π representing the total planar angular range).

In three dimensions, for a spherical Fermi surface of radius k$_F$, there are $2 \times 1/3 \times \frac{4\pi}{\lambda_F^2} = \frac{2}{3\pi} k_F^2$ conducting modes. Here, 2 is for spin degeneracy, $\frac{1}{3}$ is for averaging a vector in three dimensions, and 4π represents the solid angle. Therefore:

$$\sigma^{3D} = \frac{2}{3\pi}\frac{e^2}{h}k_F^2\ell \tag{S3}$$

Note that both these expressions for electrical conductivity are strictly equivalent to the Drude formula: $\sigma = \frac{ne^2\tau}{m}$.

To find an equivalent expression for thermal conductance, it is sufficient to multiply the expression for σ^{3D} by the Sommerfeld value $L_0 = \frac{\pi^2}{3}\frac{k_B^2}{e^2}$ leads to :

$$\frac{\kappa^{3D}}{T} = \frac{2\pi}{9}\frac{k_B^2}{h}k_F^2\ell \tag{S4}$$

This is the equation 2 of the main text.

Supplementary Note 5: Phonon dispersion in normal liquid ^{3}He.

Albergamo *et al.* used inelastic X-ray scattering to study the elementary excitations of normal liquid ^{3}He at 1.1 K. They found that the zero sound wave remains well defined in the entire range of explored wave numbers (0.15$\leq Q \leq$ 3.15 Å^{-1}) [6, 7].

Fig. shows the dispersion curve taking into account the attenuation of the wave. One can see that the energy of the sound mode shows a deep minimum near $Q = 2k_F$ [7, 10].

Krotscheck and Panholzer [11] performed a theoretical analysis of this data and found that "pair fluctuations" sharpen this mode in contrast to the random phase approximation (RPA). According to their conclusion, the collective mode has a dispersion similar to the phonon-maxon-roton dispersion of ^{4}He. The minimum was found to be close to the experimental value of 1.6 Å^{-1}.

Supplementary Note 6: The Bridgman formula and the thermal conductivity in classical liquids, glasses and quantum liquids

The Bridgman formula was written a century ago [12]. It relates the thermal conductivity to the sound velocity and particle concentration of dense liquids:

$$\kappa = rk_{\rm B}n^{2/3}v_s \tag{S5}$$

Different authors assume either $r = 3$ [13] or $r = 2$ [14, 15]. The specific heat of liquids at low temperature is close to the Dulong-Petit value, $3k_{\rm B}$, and decreases to $2k_{\rm B}$ at high temperature [16], corresponding to $r = 3$ and $r = 2$, respectively. The Bridgman formula yields a surprisingly good account of available data in liquids. The thermal conductivity of water is in agreement with $r \simeq 3$. For ethanol, methanol, butane, and pentane, the experimentally measured data agrees with using $r = 2$ with a margin of 20 percent [14].

In the case of glasses, Cahill, Watson and Pohl [17] derived a model of the minimum of thermal conductivity following an idea originally proposed by Einstein according to which thermal energy is transported by harmonic interactions between vibrating atoms with random phases. The derived expression for amorphous solids in the high field temperature limit becomes [18]:

$$\kappa_G = \left(\frac{\pi}{48}\right)^{1/3} n^{2/3} k_{\rm B}(v_l + 2v_t) \tag{S6}$$

Assuming equality between longitudinal, v_l, and transverse, v_t, phonon velocities, that is $v_l = v_t = v_s$, this equation becomes identical to the Bridgman formula, albeit with $r = 1.21$ [14, 15].

Like the Bridgman formula [12] and the asymptotic case of the Cahill-Pohl equation [17, 18], our equation 4 includes $n^{2/3}$ and $v_{th} = \sqrt{\frac{2k_BT}{m}} = \frac{v_F}{\sqrt{\pi}}\frac{\lambda_F}{\Lambda}$ rather than the sound velocity. An insight to this difference is provided by comparing the entropy production rate, which is proportional to the thermal conductivity at constant temperature and temperature gradient. In a classical system, the time scale for entropy production rate is set by the ratio of the interatomic distance divided by the sound velocity. In contrast, in a Fermi-Dirac distribution, the fundamental time scale is the ratio of the Fermi velocity to the de Broglie thermal length. As a consequence, the relevant group velocity becomes temperature dependent.

Note that in a Fermi liquid, the sound velocity v_s and the Fermi velocity v_F are intimately linked [19]:

$$v_s^2 = \frac{1}{3}(1 + F_0^s)(1 + \frac{1}{3}F_1^s)v_F^2 \tag{S7}$$

Here, F_0^s and F_1^s are Landau parameters. When $\Lambda_F = \Lambda$ at the quantum/classical crossover, we have $\mathrm{v}_{th} \approx \mathrm{v}_s$ and our equation becomes yet another version of the classical Bridgman formula.

SUPPLEMENTARY REFERENCES

[1] Meyer, H. Nuclear-Spin Exchange Interaction in Solid ^{3}He. *Journal of Applied Physics* **39**, 390–396 (1968).

[2] Greywall, D. S. Specific heat of bcc ^{3}He. *Phys. Rev. B* **15**, 2604–2623 (1977). URL https://link.aps.org/doi/10.1103/PhysRevB.15.2604.

[3] Trachenko, K., Baggioli, M., Behnia, K. & Brazhkin, V. V. Universal lower bounds on energy and momentum diffusion in liquids. *Phys. Rev. B* **103**, 014311 (2021). URL https://link.aps.org/doi/10.1103/PhysRevB.103.014311.

[4] Trachenko, K. & Brazhkin, V. V. Minimal quantum viscosity from fundamental physical constants. *Science Advances* **6**, eaba3747 (2020).

[5] Trachenko, K. Properties of condensed matter from fundamental physical constants. *Advances in Physics* **70**, 469–512 (2023).

[6] Albergamo, F., Verbeni, R., Huotari, S., Vankó, G. & Monaco, G. Zero sound mode in normal liquid ^{3}He. *Phys. Rev. Lett.* **99**, 205301 (2007). URL https://link.aps.org/doi/10.1103/PhysRevLett.99.205301.

[7] Albergamo, F., Verbeni, R., Huotari, S., Vankó, G. & Monaco, G. Albergamo et al. reply:. *Phys. Rev. Lett.* **100**, 239602 (2008). URL https://link.aps.org/doi/10.1103/PhysRevLett.100.239602.

[8] Behnia, K. On the dynamic distinguishability of nodal quasi-particles in overdoped cuprates. *SciPost Phys.* **12**, 200 (2022). URL https://scipost.org/10.21468/SciPostPhys.12.6.200.

[9] Behnia, K. On the origin and the amplitude of T-square resistivity in Fermi liquids. *Annalen der Physik* **534**, 2100588 (2022).

[10] Schmets, A. J. M. & Montfrooij, W. Comment on "zero sound mode in normal liquid ^{3}He". *Phys. Rev. Lett.* **100**, 239601 (2008). URL https://link.aps.org/doi/10.1103/PhysRevLett.100.239601.

[11] Krotscheck, E. & Panholzer, M. Theoretical analysis of neutron and X-ray scattering data on ^{3}He. *Journal of Low Temperature Physics* **163**, 1–12 (2011).

[12] Bridgman, P. W. The thermal conductivity of liquids under pressure. *Proceedings of the American Academy of Arts and Sciences* **59**, 141–169 (1923). URL http://www.jstor.org/stable/20026073.

[13] Bird, R., Stewart, W. & Lightfoot, E. *Transport Phenomena* (J. Wiley, 2002).

[14] Zhao, A. Z., Wingert, M. C., Chen, R. & Garay, J. E. Phonon gas model for thermal conductivity of dense, strongly interacting liquids. *Journal of Applied Physics* **129**, 235101 (2021). URL https://doi.org/10.1063/5.0040734.

[15] Xi, Q. *et al.* A ubiquitous thermal conductivity formula for liquids, polymer glass, and amorphous solids. *Chinese Physics Letters* **37**, 104401 (2020). URL https://dx.doi.org/10.1088/0256-307X/37/10/104401.

[16] Trachenko, K. & Brazhkin, V. V. Collective modes and thermodynamics of the liquid state. *Rep. Prog. Phys.* **79**, 016502 (2016).

[17] Cahill, D. G., Watson, S. K. & Pohl, R. O. Lower limit to the thermal conductivity of disordered crystals. *Phys. Rev. B* **46**, 6131–6140 (1992). URL https://link.aps.org/doi/10.1103/PhysRevB.46.6131.

[18] Xie, X. *et al.* High and low thermal conductivity of amorphous macromolecules. *Phys. Rev. B* **95**, 035406 (2017). URL https://link.aps.org/doi/10.1103/PhysRevB.95.035406.

[19] Vollhardt, D. & Wolfle, P. *The superfluid phases of helium 3* (CRC Press, 1990).
